# Evolutionary relationships and divergence times among the native rats of Australia

**DOI:** 10.1186/1471-2148-10-375

**Published:** 2010-12-02

**Authors:** Judith H Robins, Patricia A McLenachan, Matthew J Phillips, Bennet J McComish, Elizabeth Matisoo-Smith, Howard A Ross

**Affiliations:** 1 Department of Anthropology and School of Biological Sciences, The University of Auckland, Auckland, New Zealand; 2 Institute of Molecular Biosciences, Massey University, Palmerston North, New Zealand; 3 Research School of Biology, Australian National University, Canberra, Australia; 4 Allan Wilson Centre for Molecular Ecology and Evolution and Institute of Molecular Biosciences Massey University, Palmerston North, New Zealand; 5 Allan Wilson Centre for Molecular Ecology and Evolution and Department of Anatomy and Structural Biology, Otago School of Medical Sciences, Dunedin, New Zealand; 6 Allan Wilson Centre for Molecular Ecology and Evolution, Bioinformatics Institute and School of Biological Sciences, The University of Auckland, Auckland, New Zealand

## Abstract

**Background:**

The genus *Rattus *is highly speciose and has a complex taxonomy that is not fully resolved. As shown previously there are two major groups within the genus, an Asian and an Australo-Papuan group. This study focuses on the Australo-Papuan group and particularly on the Australian rats. There are uncertainties regarding the number of species within the group and the relationships among them. We analysed 16 mitochondrial genomes, including seven novel genomes from six species, to help elucidate the evolutionary history of the Australian rats. We also demonstrate, from a larger dataset, the usefulness of short regions of the mitochondrial genome in identifying these rats at the species level.

**Results:**

Analyses of 16 mitochondrial genomes representing species sampled from Australo-Papuan and Asian clades of *Rattus *indicate divergence of these two groups ~2.7 million years ago (Mya). Subsequent diversification of at least 4 lineages within the Australo-Papuan clade was rapid and occurred over the period from ~ 0.9-1.7 Mya, a finding that explains the difficulty in resolving some relationships within this clade. Phylogenetic analyses of our 126 taxon, but shorter sequence (1952 nucleotides long), *Rattus *database generally give well supported species clades.

**Conclusions:**

Our whole mitochondrial genome analyses are concordant with a taxonomic division that places the native Australian rats into the *Rattus fuscipes *species group. We suggest the following order of divergence of the Australian species. *R. fuscipes *is the oldest lineage among the Australian rats and is not part of a New Guinean radiation. *R. lutreolus *is also within this Australian clade and shallower than *R. tunneyi *while the *R. sordidus *group is the shallowest lineage in the clade. The divergences within the *R. sordidus *and *R. leucopus *lineages occurring about half a million years ago support the hypotheses of more recent interchanges of rats between Australia and New Guinea. While problematic for inference of deeper divergences, we report that the analysis of shorter mitochondrial sequences is very useful for species identification in rats.

## Background

*Rattus *is the most speciose genus among mammals with some 66 described species [1]. The genus is thought to have originated in the general region of the Indonesian Islands with subsequent dispersal into continental Asia, New Guinea and Australia [2]. Over the last five million years (My) a combination of tectonic and climatic change meant that this geographic region experienced major fluctuations in sea level and in the extent of emergent land mass [3]. These changes provided opportunities for dispersal and speciation within *Rattus*.

Previous studies [4,5] based on mitochondrial DNA (mtDNA) identified two major groups within *Rattus*. These are the Asian group, comprising rats from Southeast Asia and Island Southeast Asia, and the Australo-Papuan group comprising rats from Australia and New Guinea. The Asian group includes the three most widely distributed rat species, *Rattus rattus *and *Rattus norvegicus *which were dispersed around the world via European sailing ships [6] and *Rattus exulans *which was transported throughout the Pacific by prehistoric peoples [7]. The Australo-Papuan group of rats are largely restricted to Australia and New Guinea with the exception of *Rattus praetor *that also occurs in the Solomon Islands in addition to New Guinea. In addition subfossil remains of this rat have been found at archaeological sites as far east as Fiji indicating that prehistoric peoples in all likelihood transported it into the Pacific [8]. While the Robins et al. [4] study shows good species resolution among the rats of Australia the deeper relationships were not well resolved. The focus of this investigation is to use mtDNA, including whole mitochondrial genomes, to help evaluate these evolutionary relationships and to date the divergences among the *Rattus *species of Australia.

*Rattus* has been included in other recent molecular phylogenetic analyses. Jansa et al. [9] investigated relationships among endemic rodents in the Philippine Islands, using single nuclear (exon 1 of IRBP) and mitochondrial (cytochrome *b *(cyt *b*)) markers, but they included only one species from the Australo-Papuan group. Rowe et al. [10] sampled broadly among the genera of murine rodents with the aim of inferring relationships among the "Old Endemics" of the Australo-Papuan, or Sahul, region. Their use of six nuclear (exon 10 of GHR, exon 11 of BRCA1, the single large exon of RAG1, intron 3 and flanking regions of BDR, exon 1 of IRBP and intron 2 and flanking regions of ATP5) and six mitochondrial markers (the genes cytochrome c oxidase subunit I (COI), cytochrome c oxidase subunit II (COII), ATPase 8 and cyt *b*, plus the two tRNA's between COI and ATPase 8) enabled the resolution of deep divergences but their inclusion of only three *Rattus *species means that there is little overlap with our study. Most recently Pagès et al. [11] developed a large dataset of *Rattus *and related species based on one nuclear (exon 1 of IRBP) and two mitochondrial markers (cyt *b *and COI). That study, however, focussed on the species found in Southeast Asia and did not include any of the species endemic to the Australo-Papuan region.

There has been considerable debate regarding the number of rat species in Australia and New Guinea and the relationships among them. Taylor and Horner [12] revised the systematics of the native Australian rats and recognized five species *R. fuscipes*, *R. leucopus*, *R. lutreolus, R. sordidus *and *R. tunneyi*. Based on a multivariate analysis of craniometric data Taylor et al. [13] included *R. fuscipes *and *R. leucopus *within a clade containing New Guinean native rat species whereas the other Australian native rat species (*R. sordidus*, *R. tunneyi *and *R. lutreolus*) formed a separate but deeper clade. A phylogeny based on isozyme electrophoresis of 55 loci of the Australian *Rattus *species, together with *R. norvegicus *and *R. rattus *as outgroups, [14] showed similarities with the phenogram of Taylor et al. [13] with *R. lutreolus *diverging early, followed by *R. tunneyi *and lastly a more recent divergence of a clade containing *R. sordidus*, *Rattus colletti *and *R. villosissimus*. This study was unable to resolve the deeper relationships among *R. fuscipes, R. lutreolus *and *R. leucopus *and since it did not include rats from New Guinea it could not address the relationship between *R. fuscipes *and the New Guinean rats.

In the recent taxonomic study by Musser and Carleton [1] the rats of Australia and New Guinea are divided into two groups. The *Rattus fuscipes *species group comprises four of the five Australian native rats described by Taylor and Horner [12] while the fifth, *R. leucopus*, is placed among the New Guinean species in the *Rattus leucopus *species group. Musser and Carleton [1] and Baverstock et al. [14] give *R. colletti *and *R. villosissimus *full species status whereas Taylor and Horner had classified them both as sub-species of *R. sordidus*. The *Rattus leucopus *species group of Musser and Carleton [1] comprises the 14 species; *Rattus arfakiensis, Rattus arrogans, Rattus giluwensis, Rattus jobiensis, R. leucopus, Rattus mordax, Rattus niobe, Rattus novaeguineae, Rattus omichlodes, R. praetor, Rattus richardsoni, Rattus steini, Rattus vandeuseni *and *Rattus verecundus*. Of the Australian and New Guinean species, only two are known to occur in both regions and the results of the craniometric study of Taylor et al. [13] strongly supported the hypothesis of Tate [15,16] that *R. sordidus *colonised southern and south-eastern New Guinea from Australia, and *R. leucopus *colonised Cape York in north-eastern Australia from New Guinea. The presence of ephemeral land bridges between Australia and New Guinea, especially in the area of the Torres Strait, is believed to be associated with glaciation cycles during the last 2.6 - 3 million years and would have provided opportunities for such faunal interchange [17,18]. Aplin (quoted by Musser and Carleton [1] pg 1462), suggested on the basis of preliminary cyt *b *data that "members of the *R. fuscipes *and the *R. leucopus *groups each may represent discrete radiations, albeit closely related and with some exchange across the Torres strait." Aplin's [17] schematic phylogeny, although it included some New Guinean *Rattus *species, was similar to that of Baverstock et al. [14]. Aplin placed *R. lutreolus *basal in the clade containing *R. tunneyi*, *R. sordidus*, *R. colletti *and *R. villosissimus *and did not resolve the position of *R. fuscipes*. He stated that although both positions were uncertain *R. fuscipes *was probably more closely related to the New Guinean rat cluster and *R. lutreolus *to the Australian rat cluster. It is thus fair to say that there is a need for further clarification of the relationships within this speciose group.

In an earlier study Robins et al. [4] used three mitochondrial regions (D-loop, cyt *b *and COI) to identify *Rattus *species. Several well-defined clades were recovered. In many cases these corresponded to named species, that is all members of the clade had the same species label assigned at source by either the collector or the museum. In some cases, clades contained multiple species labels, possibly resulting from misidentification, faulty taxonomy or biological processes such as introgression. All 16 clades were assigned a name, as shown in Figure two of Robins et al. [4], effectively making them operational taxonomic units (OTUs) although 10 of these names corresponded well to a species. These OTUs are also used in this paper.

From the sample base of the OTUs in Robins et al. [4] six rats were chosen for whole mitochondrial genome analysis and together with data from GenBank divergence times and relationships among five *Rattus *species were inferred [5]. That 2008 study, however, included only one rat species from the Australo-Papuan region. To increase our understanding of the relationships among the rats of this region we sequenced seven whole mitochondrial genomes from six *Rattus *species from the Australo-Papuan clade; *R. fuscipes*, *R. lutreolus*, *R. sordidus*, *R. tunneyi*, *R. villosissimus *and two samples of *R. leucopus *(one originating from Australia and one from New Guinea). We have again chosen rats from the sample base of Robins et al. [4] except for the more recently acquired samples of *R. lutreolus *and *R. villosissimus*. We have sequenced whole mitochondrial genomes from four species unique to Australia and two occurring in both Australia and New Guinea. We used these data together with data from GenBank to estimate divergence times and to examine the evolutionary relationships among the Australian rats. We have compared the phylogenies inferred from whole mitochondrial genomes (n = 16) with those obtained by analysing the hypervariable region of the D-Loop together with two mitochondrial gene regions (cyt *b *and COI) from the same sample set and also from a much larger sample set (n = 126). Here we show that the use of this subset of the mitochondrial genome, while generally reliable for species identification is inadequate for dating or resolving deeper relationships. Our analyses of 16 whole mitochondrial genomes, on the other hand, have made a substantial contribution to our understanding of the relationships among the Australo-Papuan *Rattus *species and the timing of their divergences.

## Methods

Whole mitochondrial genomes were obtained from seven rats representing six different species (see sample details in Table 1). The species were *R. fuscipes*, *R. leucopus, R. lutreolus, R. sordidus, R. tunneyi*, and *R. villosissimus*. Two specimens of *R. leucopus *(one from New Guinea and the other from Australia) were processed because this species occurs in both New Guinea and in the Cape York region of Australia. These seven novel genomes were compared with other available whole mitochondrial genomes from wild caught rats. There are a number of mitochondrial genomes available from highly inbred strains of *R. norvegicus *(e.g., 10 of the 12 genomes from Schlick et al. [19]) which we did not include in the analysis. DNA was extracted from an additional two samples of *R. lutreolus *and three samples of *R. villosissimus *(see sample details in Table 2). Three mitochondrial regions were sequenced from these samples (750 bp of COI, 762 bp of cyt *b *and 585 bp of D-loop) and analysed together with the dataset from Robins et al. [4].

**Table 1:** Whole genomes used in this study.

Species	Origin	Source	Specimen Accession	GenBank Accession
*Microtus kikuchii *(vole)	Taiwan	GenBank		AF348082
*Mus musculus domesticus *(mouse)	western Europe	GenBank		NC_006914
*R. exulans*	New Zealand	EM	RNZAwa01	EU273711
*R. exulans*	Thailand	SAM	ABTC 8480	EU273710
*R. exulans*	Papua New Guinea	SAM	ABTC 43078	EU273709
R. fuscipes*	Western Australia, Australia	SAM	ABTC 8615	GU570664
R. leucopus*	Central Highland, Papua New Guinea	SAM	ABTC 42808	GU570660
R. leucopus*	Queensland, Australia	SAM	ABTC 51766	GU570659
R. lutreolus*	Tasmania, Australia	SAM	ABTC 51762	GU570661
*R. norvegicus*	Denmark	GenBank		AJ428514
*R. norvegicus*	USA	GenBank		DQ673916
*R. norvegicus*	Japan	GenBank		DQ673917
*R. praetor*	Papua New Guinea	SAM	ABTC 44065	EU273708
*R. rattus*	New Zealand	EM	RNZTitRr01	EU273707
R. sordidus*	Northern Territory, Australia	SAM	ABTC 41164	GU570665
*R. tanezumi*	Japan	SAM	ABTC 8514	EU273712
R. tunneyi*	Northern Territory, Australia	SAM	ABTC 29636	GU570662
R. villosissimus*	South Australia, Australia	SAM	ABTC 00549	GU570663

An * indicates the genomes sequenced as part of this study. EM = Lisa Matisoo-Smith, SAM = South Australia Museum

**Table 2:** Additional sequences for the three genomic regions.

			GenBank Accession		
Species	Origin	Specimen Accession	COI	Cyt *b*	D-loop
*R. lutreolus*	Myall Lakes, New South Wales	ABTC 51720	GU570676	GU570671	GU570666
*R. lutreolus*	Jervois, South Australia	ABTC 27458	GU570677	GU570672	GU570667
*R. villosissimus*	Purni Bore, South Australia	ABTC 00548	GU570678	GU570673	GU570668
*R. villosissimus*	Palparara, south west Queensland	ABTC 23632	GU570679	GU570674	GU570669
*R. villosissimus*	Sir Edward Pellew Is., Northern Territory	ABTC 41137	GU570680	GU570675	GU570670

All specimens were collected in Australia. Tissue samples were provided from specimens by the South Australia Museum.

DNA was extracted from liver or muscle tissue using the High Pure PCR Template Preparation Kit (Roche). Mitochondrial DNA was amplified in four overlapping long range pieces from 4 to 6 kb in length. See Table 3 for amplicon length and primer details. Long range PCR was performed using the DNA polymerase Taq and protocols of the Expand Long Template PCR System (Roche). For short-range PCR the amplification reactions contained: TrisHCl pH 8.3 10 mM; KCl 50 mM; forward and reverse primers at 0.5 μM each; dNTPs at 0.15 mM each; 0.5 U of Taq polymerase; 1 μL of DNA template. We used a standard 3-step amplification protocol: 94°C, 3 min, 35 cycles of: 94°C, 30 s, 60°C, 30 s, 72°C, 1 min followed by 1 cycle of 72°C, 5 min and a hold at 15°C. PCR products were visualized and quantified on ethidium bromide stained 1% agarose gels using a low mass ladder from Invitrogen for comparison and purified using either ExoSAP-IT™ from USB Corporation or band cut and column cleaned using the QIAquick Gel Extraction Kit™ from QIAGEN.

**Table 3:** The primers used to amplify the four overlapping long range amplicons.

A.		
**Primer name**	**Sequence (5' to 3')**	

Av175312SF	AAACTGGGATTAGATACCCCACTAT	
R6036R	ACTTCTGGGTGTCCAAAGAATCA	
BatL5310	CCTACTCRGCCATTTTACCTATG	
LR2RB	CTGATTGGAAGTCAGTTGTATTTTT	
L10647F	TTTGAAGCAGCAGCCTGATAYTG	
RCb9H	TACACCTAGGAGGTCTTTAATTG	
RGlu2L	CAGCATTTAACTGTGACTAATGAC	
Long 16SR	TGATTATGCTACCTTTGCACGGTCAGGATACC	

**B.**		

**Region amplified**	**Primer pairs**	**Amplicon length**

12S - COI	Av175312sf & R6036R	5.55 kb
COI - COIII	BatL5310 & LR2RB	4.11 kb
COIII - Cyt *b*	L10647F & RCb9H	5.53 kb
Cyt *b *- 16S	RGlu2L & Long 16SR	4.26 kb

(A) The primer sequences (5' to 3'). (B) The primer pairs used for each genome region and the amplicon length.

All sequencing was done by The Allan Wilson Centre Genome Service at Massey University, Palmerston North, New Zealand. The long range products from each sample except *R. fuscipes *which was sequenced as part of a separate experiment, described in McComish et al. [20] were pooled and processed for sequencing using the multiplexed sequencing kit from Illumina. A 75-bp single read run was performed on an Illumina Genome Analyser GAII (Illumina, Inc.) according to the manufacturer's instructions. After sequencing, the resultant images were analysed with the proprietary Illumina pipeline (version 1.4). This resulted in approximately 964 Mb of sequence, with 90% of clusters passing the initial filtering step. Reads for each sample were trimmed at the 3' end by 5, 10, 15 and 20 bases. Assemblies were made using Velvet 0.7 [21] with a range of hash lengths from 33 to 63 and a minimum k-mer coverage of 5×. Maximum contig lengths and N50 values were tabulated, and the best assembly for each sample was selected for further analysis. Assembled contigs were aligned to the *R. praetor *mitochondrial genome using Geneious 4.7 [22].

Sanger sequencing was used to sequence both ends of each long range fragment to check consistency and to complete the coverage of the mitochondrial genomes. It was also used to sequence the three mitochondrial regions of the additional samples of *R. lutreolus *and *R. villosissimus*. Sanger sequencing was carried out using the BigDye Terminator version 3 sequencing kit, the GeneAmp PCR System 9700 and a capillary ABI3730 DNA Analyser, all from Applied Biosystems. The final assembly of each mitochondrial genome was carried out in Sequencher™ (Gene Codes Corporation).

### Sequence Alignments

Three main datasets were assembled. The whole genome (WG) dataset (for the alignment see Additional File 1) contained mitochondrial DNA sequences from the genomes of 13 species and included the vole (Cricetidae) and mouse (Muridae) as outgroups (Table 1). The dataset includes 12 mitochondrial genome proteins, rRNA and tRNA coding sequences, totalling 14,471 nucleotides after manual alignment. The D-loop was excluded because of difficulty in alignment. The sequence for protein coding gene ND6 was also excluded because it is encoded on the mitochondrial L-strand. The sequence alignment was partitioned into the three codon positions, RNA stem regions and RNA loop regions. As in the previous work of Robins et al. [5], the third codon positions were coded as purines or pyrimidines (RY-coded) because it was demonstrated there that these positions had a higher substitution rate than the other partitions and substitution models did not sufficiently account for saturation at these sites at deeper levels in the tree. RY-coding greatly reduced saturation in 3^rd ^codon transitions, removed highly significant composition bias and provided better phylogenetic resolution, even within *Rattus *[5]. Compositional heterogeneity is a recognised problem in mitochondrial genome analysis and for a more detailed discussion of the issues see [23] and other references therein.

A second alignment was assembled from three genomic regions (3G), comprising 126 sequences, spanning 672 bp of cyt *b*, 702 bp of the COI and the hypervariable region of the D-loop, for a total length of 1952 bp. This dataset which includes *Mus *as the outgroup extends that of Robins et al [4] by the inclusion of sequences from seven new samples, three of *R. lutreolus *and four of *R. villosissimus *(for sample details see Tables 1 and 2). The vole outgroup was not included because of the difficulty in aligning the D-loop. This alignment was analysed in three ways: with three partitions (codon positions 1+2, codon position 3 as RY, and all other positions see Additional File 2), with each genomic region as a separate but unencoded partition (Additional File 3), and as a concatenated sequence with no partitions. The coding of sequence labels in the nexus files to species names and GenBank accessions is given in Additional File 4.

A third alignment (3G-WG) was created (Additional File 5) by extracting the 3G regions from the specimens in the WG dataset. This enabled us to assess whether the differences in the results obtained using the WG and 3G datasets were due to the specimens or to the genomic regions used.

Of the gene regions that we have sequenced, cytochrome *b *is the most widely represented among rodent species in GenBank. To test whether the Australo-Papuan clade was monophyletic we assembled a rodent alignment of cytochrome *b *sequences from 245 representatives of *Rattus sensu lato *[24] (see Additional File 6). This included our data as well as additional data deposited in GenBank by Pagès et al. [11], Jansa et al. [9], and Rowe et al. [10]. The sequences from the various studies tended to be either ~700 or ~1200 bp in length. Further we wished to use both unencoded and RY-encoded data. Consequently we created four alignments. The first used the complete alignment from Additional File 6 (1-1182 bases), and the second used a short alignment (bases 1-713). In the third and fourth alignments the data from both the complete and the short alignment were partitioned into codon positions 1+2 (unencoded) and codon position 3 (coded as RY). These codon-partitioned alignments were trimmed by 40 bases at the 3' end to remove the non-coding tRNA region from the sequences leaving bases 41-1182 and 41 - 713 respectively.

### Phylogenetic methods

The optimal substitution model was determined in previous work [5] to be GTR + I + G_4_. This model was used in partitioned maximum likelihood (ML) and Bayesian inference analyses. ML analyses were performed on the WG dataset with PAUP* [25] and RAxML [26]. Other exploratory ML analyses were performed using PHYML [27]. Phylogenetic trees were estimated from the WG, 3G, 3G-WG and the four rodent cytochrome *b *alignments under Bayesian inference with MrBayes [28]. For the WG dataset, three Markov chain Monte Carlo (MCMC) chains for each of two independent runs proceeded for 8,500,000 generations with trees being sampled every 5000 generations. The burnin length 1,500,000 was determined by examination of -ln*L *versus MCMC generation plots and sampling efficiency within Tracer 1.5 [29]. For the 3G and 3G-WG datasets, three MCMC chains for each of two independent runs were sampled for 10,000,000 generations with trees sampled every 1000 generations. The burnin length of 100,000 generations was chosen by the same method.

In addition to the three phylogenetic trees estimated from each of the WG, 3G and 3G-WG datasets, estimations were made using ML for alignments based on single gene regions from both the WG and 3G datasets. In all, 18 tree topologies were obtained for the species represented in the WG dataset. Support in the WG dataset among these alternative topologies was examined using the Kishino-Hasegawa (KH) [30] and approximately unbiased (AU) [31] tests within the CONSEL program [32]. Sitewise log likelihoods were combined from the GTR + I + G_4 _(CF87 + I + G_4 _for RY-coded data) maximum likelihood (ML) analyses run in PAUP* for each of the separately run partitions.

**Figure 1:**
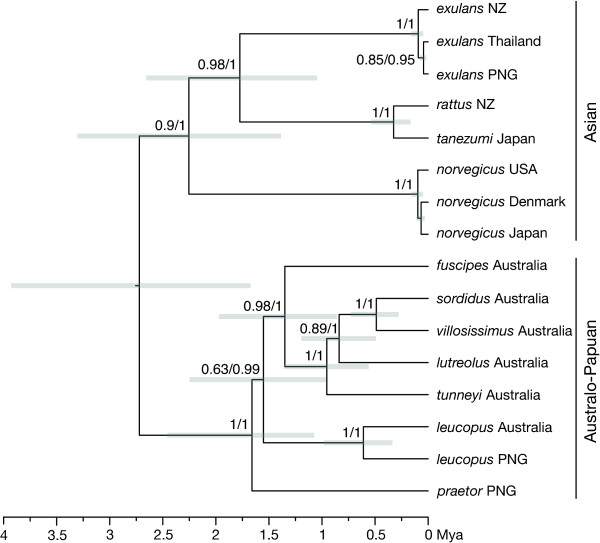
**Bayesian Inference chronogram from BEAST estimated for the whole genome (WG) dataset**. The grey bars represent the 95% credible intervals for tMRCAs for *Rattus *species from BEAST. The ML bootstrap support (BS) and the Bayesian posterior probability (BPP) is given at each node (BS/BPP). The vole and mouse outgroups have been removed to improve legibility.

**Figure 2:**
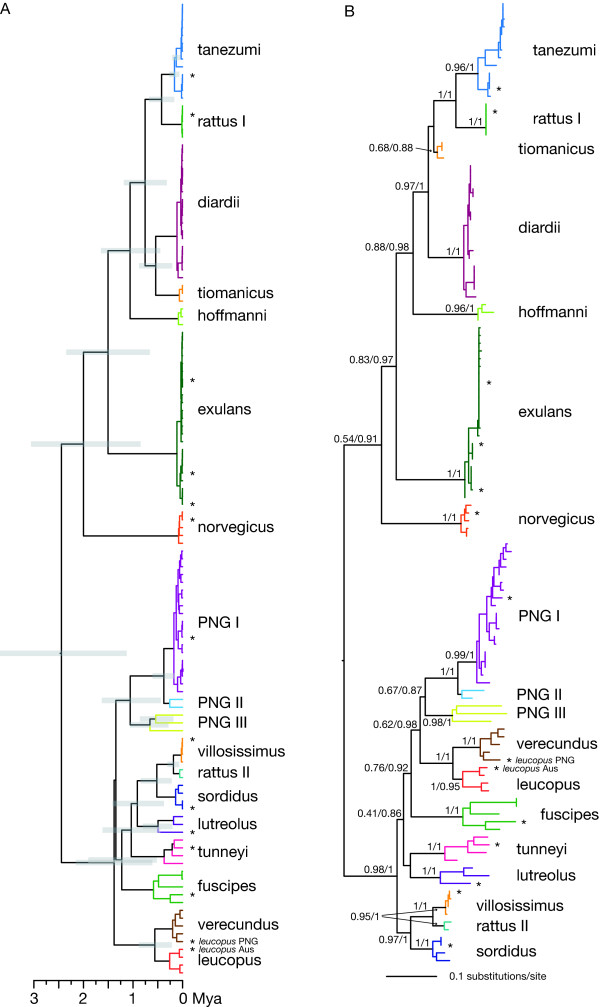
**(A) Bayesian Inference chronogram and (B) ML and MrBayes tree estimated for the three genome region (3G) dataset**. Clades are individually colored, and labelled according to the "phylogenetic species" identified in Robins et al [4]. The grey bars represent the 95% credible intervals for tMRCAs for *Rattus *clades from BEAST. In the BEAST tree (A), the outgroup has been removed, the Asian and the Australo-Papuan clades are constrained to be monophyletic and a prior is assigned to the root age as described in the text. The ML and MrBayes tree (B) is labelled with bootstrap support (BS) and the Bayesian posterior probability (BPP) at each node (BS/BPP). The mouse outgroup has been removed to improve legibility. Sequences marked with * are from the WG dataset.

We assessed variation along the genome in the strength of support for the two main topologies, that with the clade comprising *R. lutreolus*, *R. sordidus*, *R. tunneyi *and *R. villosissimus *(Figure 1) or that with the clade comprising *R. fuscipes*, *R. leucopus *and *R. praetor *(Figure 2B) as a more recent divergence. The whole genome alignment was subdivided into 1000 bp sections, and an ML bootstrap analysis performed on each non-partitioned section using PHYML and the GTR+G+I model of evolution. We estimated the frequency of each clade for each region among the bootstrap trees.

BEAST [33] was used to apply Bayesian inference to the estimation of divergence dates within the phylogenies of the WG, 3G and 3G-WG species. For the WG dataset, as in previous work [5], a partitioned analysis was performed in which codon positions 1 and 2 were combined, RNA stem and loop partitions were combined and codon position 3 was coded as RY, to counteract the effects of rate heterogeneity and substitution saturation at hypervariable sites. The MCMC chain was run for 10,000,000 generations, with both trees and ages sampled every 5,000 generations. The "relaxed" clock method was used in which rates along branches are distributed according to a lognormal distribution [34]. For the 3G and 3G-WG datasets, a partitioned analysis was performed in which codon positions 1 and 2 were combined, codon position 3 was coded as RY and all other sites formed the third partition. A birth-death model of speciation was used with the WG and 3G-WG datasets, in which each species is represented by a single sequence, whereas a coalescent model was used with the 3G dataset, in which each species has multiple specimens. The sampled trees were summarised as maximum clade credibility trees with node heights set to the median of sampled values using TreeAnnotator [33].

We applied age constraints on two nodes to calibrate our BEAST analysis of the WG dataset: the split between Muridae (mouse plus rats) and Cricetidae (vole), that is the root node, and the split between *Mus *and *Rattus *within Muridae. As in previous work [5] we used the recommendations of Benton and Donoghue [35] for the minimum and maximum age constraints of the mouse-rat divergence. They state that current research suggests that the split between *Mus *and *Rattus *is early in the evolution of Murinae but not basal in the divergence of the clade. They recommend a lower bound of 12.3 Mya since this is the oldest record of *Progonomys*, the genus assumed to contain the common ancestor of *Mus *and *Rattus *and an upper bound of 11 Mya which is based on records of the extinct genus *Karnimata *which is believed to be on the lineage leading to *Rattus*. These bounds were used respectively for the middle 95% of the normally distributed prior. The *Mus *plus *Rattus *clade was enforced relative to the vole which is in agreement with all recent molecular and morpological interpretations. A uniform prior from 11 to 34 Mya for Muridae (mouse and rats) versus Cricetidae (vole) provided a calibration on the root that is very conservative at both the upper and lower bounds (see [36]).

The chronogram obtained (as described above) from BEAST for the 3G dataset contained unrealistically old ages for the nodes in the tree and a topology that differed markedly from that estimated for the WG dataset. Consequently, to test the effect of substitutional saturation, a further BEAST analysis was performed using a coalescent prior and constant population size. Here, the *Mus *outgroup was removed because of the long branch lengths between *Mus *and *Rattus *and potential saturation in the hypervariable region of the D-loop. The Asian and Australo-Papuan clades were each constrained to be monophyletic and the age of the root, the age of the most recent common ancestor (tMRCA) for *Rattus*, was assigned a lognormal prior based on the empirical results from the WG analysis.

## Results

### Topology

Three datasets were used for phylogenetic reconstructions. These differed in their extent of taxon sampling and in their sequence length (whole mitochondrial genomes (WG): 16 taxa 16293-16309 characters; three genomic regions (3G): 126 taxa 1952 characters; whole mitochondrial genomes trimmed to three regions (3G-WG): 16 taxa 1952 characters). As described below, similar but different topologies were inferred for relationships among the Asian clade when the larger taxon dataset was used. Differences in the relationships among Australian species were also inferred when New Guinean sequences were included in the analysis of Australian species. The greatest uncertainty in relationships among the Australian species was due to different root placements being optimal for the Australo-Papuan clade in some analyses of the different datasets. As we discuss, these observations are consistent with expectations for rapid speciation [37,38].

#### The whole genome (WG) dataset

The trees estimated by ML and Bayesian inference from the WG dataset have the same topology (Figure 1) with the minor exception of differences in the branching order among the *R. norvegicus *sequences. The topological relationships within the clade of Asian species (*R. exulans*, *R. norvegicus*, *R. rattus *and *R. tanezumi*) are the same as previously recovered [5]. The Australo-Papuan species (*R. fuscipes*, *R. leucopus*, *R. lutreolus*, *R. praetor*, *R. sordidus*, *R. tunneyi *and *R. villosissimus*) form a sister clade to the Asian clade. Overall eighteen hypotheses regarding the relationships among the Australo-Papuan clade of *Rattus *were tested using the CONSEL program. The log-likelihood differences between the optimal topology and the other hypotheses of relationship for the WG data are given in Table 4. The relationships represented in Figure 1 had the highest likelihood, but four other topologies were clearly not significantly poorer and a fifth was only marginally not significantly poorer. Common features of the five best trees include a) the early divergence of *R. praetor *and *R. leucopus *from the other lineages, and b) the late divergence of *R. sordidus *and *R. villosissimus*. Trees in which *R. praetor *diverge late (e.g. 12, 16, 18 ) are much poorer than the best trees.

**Table 4:** Log-likelihood differences between trees.

	Tree topologies	-ln*L*	*P *values	
			
			AU	KH
1	(**Out**,(**AR**,((((((Rsor,Rvil),Rlut),Rtun),Rfus),**Rleu**),Rpraet)))	<41273.6>	--	--
2	(**Out**,(**AR**,((((((Rsor,Rvil),Rlut),Rtun),Rfus),Rpraet),**Rleu**)))	2.6	0.556	0.351
3	(**Out**,(**AR**,(((((Rsor,Rvil),Rlut),Rtun),Rfus),(**Rleu**,Rpraet))))	4.1	0.393	0.254
4	(**Out**,(**AR**,(((((Rsor,Rvil),(Rlut,Rtun)),Rfus),Rpraet),**Rleu**)))	9.3	0.208	0.170
5	(**Out**,(**AR**,((((Rsor,Rvil),(Rtun,Rlut)),Rfus),(**Rleu**,Rpraet))))	11.2	0.144	0.126
6	(**Out**,(**AR**,(((((Rsor,Rvil),Rlut),Rtun),(**Rleu**,Rfus)),Rpraet)))	16.3	0.082	0.054
7	(**Out**,(**AR**,((((((Rsor,Rvil),Rlut),Rtun),**Rleu**),Rpraet),Rfus)))	25.8	0.020*	0.018*
8	(**Out**,(**AR**,(((((Rsor,Rvil),Rtun),Rlut),(Rfus,Rpraet)),**Rleu**)))	30.9	0.019*	0.018*
9	(**Out**,(**AR**,((((Rsor,Rvil),Rtun),Rlut),((**Rleu**,Rfus),Rpraet))))	31.2	0.028*	0.021*
10	(**Out**,(**AR**,(((Rsor,Rvil),(Rtun,Rlut)),((Rfus,Rpraet),**Rleu**))))	34.0	0.025*	0.013*
11	(**Out**,(**AR**,((((Rsor,Rvil),Rtun),Rlut),((**Rleu**,Rfus),Rpraet))))	101.9	>0.001*	>0.001*
12	(**Out**,(**AR**,((Rsor,Rvil),((Rlut,Rtun),(Rfus,(**Rleu**,Rpraet))))))	133.8	>0.001*	>0.001*
13	(**Out**,(**AR**,((((Rsor,Rtun),Rvil),(Rlut,Rfus)),(**Rleu**,Rpraet))))	199.0	>0.001*	>0.001*
14	(**Out**,(**AR**,(((((Rsor,Rvil),**Rleu**),Rfus),(Rtun,Rlut)),Rpraet)))	208.6	>0.001*	>0.001*
15	(**Out**,(**AR**,((((Rsor,Rvil),Rlut),Rtun),(Rfus,(**Rleu**,Rpraet)))))	274.2	>0.001*	>0.001*
16	(**Out**,(**AR**,(((((Rsor,Rvil),Rlut),Rtun),(Rpraet,Rfus)),**Rleu**)))	277.7	>0.001*	>0.001*
17	(**Out**,(**AR**,((((Rsor,Rlut),Rtun),((Rvil,Rfus),Rpraet)),**Rleu**)))	343.5	>0.001*	>0.001*
18	(**Out**,(**AR**,((Rsor,Rvil),((Rlut,Rtun),((Rfus,Rpraet),**Rleu**)))))	409.9	>0.001*	>0.001*

The relationships among Australo-Papuan *Rattus *species and their statistical significance under AU and KH tests. The column headed -ln*L *gives the log-likelihood of the best tree in the first line and the differences in log-likelihood of the other trees in subsequent lines. * indicates *P *≤ 0.05. Taxon abbreviations: Rex *R. exulans*, Rfus *R. fuscipes*, Rleu *R. leucopus*, Rlut *R. lutreolus*, Rnov *R. norvegicus*, Rpraet *R. praetor*, Rrat *R. rattus*, Rsor *R. sordidus*, Rtan *R. tanezumi*, Rtun *R. tunneyi*, Rvil *R. villosissimus*. **Out **represents the non-*Rattus *Mouse and Vole and **AR **the Asian *Rattus *clade(((**Rex**),(**Rrat**, Rtan)),(**Rnov**)). **Rex **comprises the taxa RexNZ, RexThai, RexPNG; **Rnov **comprises the taxa RnovDen, RnovUSA, RnovJap; **Rleu **comprises the taxa Rleu Australia, Rleu Papua New Guinea.

The Australo-Papuan clade has strong support from both ML and Bayesian inference (Figure 1). Within the Australo-Papuan clade, the analyses of the WG dataset indicate support for *R. sordidus *and *R. villosissimus *as sister taxa, with the inclusion of *R. lutreolus*, *R. tunneyi *and then *R. fuscipes *as one moves back in time. The order of divergence of *R. praetor *and *R. leucopus *is somewhat uncertain; the time interval between the dates of divergence is small and ML bootstrap support is weak.

#### Three mitochondrial gene (3G) region (Cyt b, COI, D-Loop) datasets

Phylogenetic analysis of the 3G dataset obtained from ML and MrBayes analyses, when the codon positions are partitioned and RY encoding used, returned the same topology (Figure 2B) corresponding to topology 12 in Table 4 where, as a member of the **PNG I **clade, *R. praetor *diverges recently and *R. lutreolus*, *R. sordidus*, *R. tunneyi *and *R. villosissimus *have more basal positions. This topology is not supported by the WG dataset (see Figure 1).

A slightly different topology again was found when the 3G dataset was analysed with MrBayes without RY encoding, either with the alignment partitioned by region or simply concatenated. Here, the order of divergence of *R. leucopus *and *R. praetor *is reversed, and *R. lutreolus *and *R. tunneyi *form a clade. This corresponds to topology 4 of Table 4.

**Figure 3:**
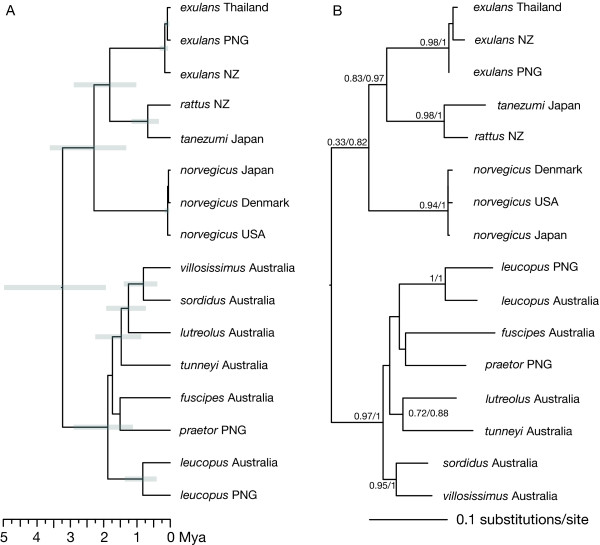
**(A) Bayesian Inference chronogram and (B) ML and MrBayes tree estimated for the 3G-WG dataset**. The 3G-WG dataset comprises the three genomic regions (COI, CYT *b *and D-loop) using the same individuals as in the WG dataset. The grey bars in the chronogram (A) represent the 95% credible intervals for tMRCAs for *Rattus *species from BEAST. The ML and MrBayes tree (B) is labelled with bootstrap support (BS) and the Bayesian posterior probability (BPP) at each node (BS/BPP). The mouse outgroup has been removed to improve legibility.

The major differences in tree topology inferred from the 3G dataset, as compared with the WG dataset, may derive from the impact of the many additional sequences in the 3G dataset or from the reduction in the number of genomic regions and partitions, and the inclusion of the hypervariable D-loop. To test this possibility, the 3G dataset was pruned of all sequences not in the WG dataset. For the tree returned by a BEAST analysis of this 3G-WG dataset (Figure 3A; topology 16 in Table 4), the unrooted topology of the Australo-Papuan clade is highly similar to that shown in Figure 1. It differs only in the position of *R. praetor*, however, the root of the Australo-Papuan clade is placed differently. Both ML and MrBayes estimations of the phylogenetic tree produced a topology (Figure 3B) with notable differences in the positions of the root of the Australo-Papuan clade and of *R. tunneyi *(topology 18 in Table 4).

**Figure 4:**
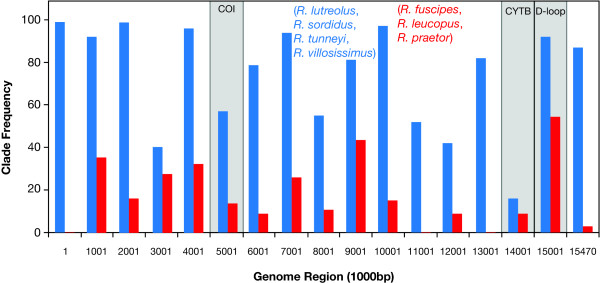
**Support for clades along the *Rattus *genome**. The bootstrap support for two clades, (*R. praetor*, *R. fuscipes *and *R. leucopus*) in a derived position (red) and (*R. lutreolus*, *R. tunneyi*, *R. sordidus*, *R. villosissimus*) in a derived position (blue) on ML trees estimated for non-overlapping 1000 bp regions of the whole genome. The sections containing the 3 genomic regions (COI, Cyt *b *and D-loop) are shaded. Note that the sum of the bootstrap proportions can exceed 100% when both clades occur in a tree as in the D-loop region of the genome.

The frequency along the genome for the two topologies, that with the clade comprising *R. lutreolus*, *R. sordidus*, *R. tunneyi *and *R. villosissimus *(Figure 1) or that with the clade comprising *R. fuscipes*, *R. leucopus *and *R. praetor *(Figure 2B) as a more recent divergence is summarized in Figure 4. The support for these clades is highly variable along the genomes. The fragment containing the D-loop provided the strongest support for the (*R. fuscipes*, *R. leucopus *and *R. praetor*) clade. Further, it contained strong support for the (*R. lutreolus*, *R. sordidus*, *R. tunneyi *and *R. villosissimus*) clade. The sections containing the COI and Cyt *b *genomic regions provided only weak support for either of these clades.

Another approach to testing the effects of the gene regions used was to remove the outgroup and constrain the height of the tree. This will reduce the possibility of misplacing the root of the Australo-Papuan clade as a result of long branch attraction relative to the outgroup and stochastic artefacts. It will also diminish the influence of hypervariable regions of the sequence. The topology returned from this analysis (Figure 2A) was very similar to, and not significantly different from, that obtained in the WG analysis (Figure 1), corresponding to topology 2, or marginally topology 1, in Table 4.

In summary, both the 3G and 3G-WG datasets gave topologies significantly different from that obtained using the WG dataset when Bayesian inference (both BEAST and MrBayes) or maximum likelihood was used to analyse partitioned and RY-coded datasets. Reducing the effects of the outgroup or the hypervariable regions enabled us to recover the WG topology. Consequently we can infer that it was the use of only these three limited genomic regions, rather than the inclusion of many additional sequences, that led to the inference of a different topology from the 3G dataset than from those of the pruned 3G-WG and the WG datasets. Nevertheless our analyses of the 3G dataset provided generally well supported species clades which are useful for species identification.

The four cytochrome *b *alignments returned very similar results. We provide the tree with species clades collapsed for greater legibility in Additional File 7 and as a full tree file in Additional File 8. In each analysis the order of divergence was *Maxomys *deepest followed by *Micromys*, *Niviventer *and *Leopoldamys*. The Australo-Papuan rats always formed a well-supported monophyletic clade. The clades representing *Bandicota*, *Berylmys*, *Tarsomys*, *Limnomys*, *R. norvegicus *and its sister species *R. nitidus*, and the remaining clade of Asian rats were each observed consistently with high support. However, the relationships among them were unstable and were not resolved with high posterior probability or bootstrap support (results not shown).

### Dates of Divergence

The addition of seven genomes has made more recent the estimated MRCAs within the *Rattus *genus when compared with previous work [5]. The median age of the split between the Asian and Australo-Papuan clades is estimated to be 2.7 Mya (95% credible interval 1.7 - 3.9 Mya). Our previous estimate of this date, based on eight Asian rat genomes but only a single genome from the Australo-Papuan clade (*R. praetor*), was near the upper bound at 3.8 Mya. Similarly, the previous estimate of tMRCA of the Asian clade, at 3.1 Mya, falls near the upper bound of the estimate obtained here with median 2.3 and 95% credible range 1.4 - 3.3 Mya. The MRCA for the Australo-Papuan clade is 1.66 Mya, and the most recent divergence within that clade, between *R. sordidus *and *R. villosissimus*, is estimated to be approximately 500,000 ya (Table 5).

**Table 5:** The estimated age of the most recent common ancestor of Rattus clades.

Clade	Median tMRCA [95% HPD]
**Australo-Papuan**	
*R. fuscipes*, *R. leucopus*, *R. lutreolus*, *R. praetor*, *R. sordidus*, *R. tunneyi *and *R. villosissimus*	1.66 [1.07 - 2.46]
*R. fuscipes*, *R. leucopus*, *R. lutreolus*, *R. sordidus*, *R. tunneyi *and *R. villosissimus*	1.55 [0.96 - 2.25]
*R. fuscipes*, *R. lutreolus*, *R. sordidus*, *R. tunneyi *and *R. villosissimus*	1.35 [0.86 - 1.97]
*R. lutreolus*, *R. sordidus*, *R. tunneyi *and *R. villosissimus*	0.95 [0.56 - 1.35]
*R. lutreolus*, *R. sordidus*, and *R. villosissimus*	0.84 [0.49 - 1.20]
*R. sordidus*, and *R. villosissimus*	0.49 [0.28 - 0.73]

Median estimates (Mya) with 95% credible limits for the MRCA of the Australo-Papuan clade of *Rattus *species and the divergences within that clade (Figure 1).

When the dates of divergence were estimated for the 3G dataset, nodes in the tree were more than twice as old as when estimated using the WG dataset. However, when the MRCA for *Rattus *was constrained, the ages of the divergences in the Australo-Papuan clade (Figure 2A) more closely resembled those obtained from the WG dataset.

The estimates of the node ages obtained using the 3G-WG dataset (Figure 3) are comparable but slightly older than those obtained from the whole genome. The Asian - Australo-Papuan split is at 3.2 Mya as compared with 2.7 Mya, and the Australo-Papuan clade has a MRCA of 1.87 Mya as compared with 1.66 Mya from the whole genome. In contrast, the deeper splits within the Asian clade have nearly the same estimated age. The divergence of *R. norvegicus *is estimated to be 2.28 Mya, as compared with 2.25 Mya, and the divergence of *R. exulans *from the other *Rattus *at 1.81 versus 1.77 Mya when the whole genome was used.

## Discussion

Whole mitochondrial genomes have been used to infer phylogenetic relationships in a wide range of organisms e.g., mammals [39-43], birds [44], fish [45] and worms [46]. Phylogenies based on mitochondrial genomes are effectively gene trees and so may not recover the true species tree [47]. While it may be useful also to develop phylogenies based on nuclear markers, there are several reasons why the use of whole mitochondrial genomes provide good indicators of species history. The mitochondrial genome, being haploid and lacking recombination, effectively has four-fold lower coalescence times relative to diploid nuclear genes. This gives much greater phylogenetic resolution than slower evolving nuclear genes. Hence mitochondrial genomes are expected to be good indicators of species history.

Our phylogeny estimated from the WG dataset (Figure 1) is well resolved except for the relative positions of *R. leucopus *and *R. praetor*. The top six observed tree topologies (Table 4) have one or the other of these two species diverging earliest in the Australo-Papuan clade. Both species diverge earlier than any of the Australian native rats and there is marginally more support for *R. praetor *diverging earliest. This phylogeny clarifies the relationships among the rats of the *Rattus fuscipes *species group and is consistent with the taxonomy of Musser and Carleton [1]. The occurrence of *R. villosissimus *and *R. sordidus *as sister species in a shallow divergence is concordant with the phenetic results of Taylor et al. [13], and the phylogenies of Baverstock [14] and Aplin [17]. The more recent divergence of *R. lutreolus *compared with *R. tunneyi *differs from these earlier phylogenies where *R. lutreolus *is either basal to *R. tunneyi *[13,17] or in an unresolved polytomy that includes *R. fuscipes *and *R. leucopus *[14]. In our phylogeny the position of *R. lutreolus *within the Australian native rat clade supports Aplin's [17] view that this rat is more closely related to Australian than to New Guinean rats. The position of *R. fuscipes *basal in our Australian native rat clade but a more recent divergence than that of *R. leucopus *differs from the results of Taylor et al. [13] whose phenogram shows *R. fuscipes *within a clade of New Guinean rats and diverging earlier than the New Guinean species *R. leucopus*.

Our choice of samples for whole mitochondrial genome analysis was influenced by our confidence in the correct assignment of species to those samples. Where there was congruence between the named species and the phylogenetic clades we were confident in the identifications. In Robins et al. [4] we discussed the difficulties that arose when OTUs contained heterogeneous collections of named species. These names were assigned by either the museum or the collector. The source of such heterogeneity could be misidentification, faulty taxonomy due to the plethora of synonyms used for *Rattus *species, hybridisation, incomplete lineage sorting or some combination of the above. Pagès et al. [11], although they explicitly avoided rats from the Australo-Papuan clade, reported a similar problem. They identified a heterogeneous clade (R3) that was similar to, and where they incorporated data from Robins et al. [4], essentially the same as the **diardii **clade (Figure 2) therein. In this paper we again resolved the **diardii **clade. When Pagès et al. [11] considered the same species as we, their genetic based species identifications are largely congruent with ours. This finding supports the usefulness of shorter mitochondrial sequences for species identification. Of the 14 *Rattus *specimens we used for the whole mitochondrial genomes, 11 were from homogeneous clades. The exceptions were the **exulans **clade which from a total of 21 samples contained two almost certainly misidentified samples and two New Guinean clades **PNG I **and **verecundus**. Our New Guinean species clades are all problematic and to resolve the conflict DNA is needed from specimens that have accurate morphological identifications. All six of the specimens of *R. praetor *that we have processed fell into the **PNG I **clade and we used one of these samples to represent the clade. At this time we have insufficient information to account for the heterogeneity of this clade although some possibilities are discussed in Robins et al. [4]. The Australian *Rattus *specimens on the other hand all fell into well supported homogeneous clades thus the phylogenetic assignment of species was in agreement with that of the museum or collector.

Our cytochrome *b *analyses generally gave robust species clades. Further, the Australo-Papuan rats were in a well-supported and monophyletic clade in all analyses. However, the relationships within *Rattus sensu stricto *[24], especially among *Bandicota*, *Berylmys*, the Philippine endemics (*Tarsomys*, *Limnomys *and *Rattus everetti*) and the main Asian and Australo-Papuan clades of *Rattus*, were poorly resolved. On the other hand, the deeper relationships within Rattus *sensu lato *were well-supported. Pagès et al. [11] stated that they did not obtain a robust phylogeny when they analysed cytochrome *b *alone. Interestingly, even when they analysed three concatenated genes (one nuclear and two mitochondrial) they were unable to place *Bandicota *conclusively within or sister to *Rattus*. Thus the currently available data is insufficient to address the question of monophyly within *Rattus *as a whole.

The ancestral node in the Australo-Papuan clade differed with the genomic regions used. The inclusion of the hypervariable D-loop (Figures 2B and 3) moved the root deeper into the clade, relative to its placement when the whole genome was used (Figure 1) or when the MRCA of *Rattus *was constrained (Figure 2A). It is likely that the differences arise from substitution rate differences between D-loop and the rest of the genome. The hypervariable nature of the D-loop, with the potential for substitution saturation, also contributed to the inflation (two- to three-fold increase) of dates of divergence in the unconstrained BEAST tree based on the 3G dataset compared with that of the WG dataset.

Although the deeper branching orders are uncertain within the Australo-Papuan clade for the 3G and the 3G-WG phylogenies (Figures 2 and 3) the Australian native *Rattus *species, including the newly added *R. lutreolus *and *R. villosissimus *samples fall into well differentiated clades reinforcing the usefulness of shorter mitochondrial DNA sequences for species identification [48]. The position of two samples from New Guinea, misidentified as *R. rattus *and labelled as OTU **rattus II **in Figures 2A and 2B, as sister to *R. villosissimus *which is in turn sister to *R. sordidus *suggests that these samples are in fact *R. villosissimus*. The usefulness of these shorter sequences, however, to elucidate deeper evolutionary relationships or to date divergences is expected to be limited given the difficulties in inferring species relationships for rapid radiations [37,38]. The deeper branches of the Australo-Papuan clade in Figures 2 and 3 are all quite short, indicative of a period of rapid diversification, but the branching pattern varies with the methods used and no one pattern is well supported. It is possible that the inclusion of one or more nuclear markers could help resolve this pattern and is an approach to be pursued in the future, however, given their greater effective population size and longer coalescence times, nuclear genes are likely to be less powerful in resolving the order of divergences. They will, however, provide evidence regarding whether mitochondrial gene trees provide accurate estimates of the deeper species relationships [e.g., [49]].

The dates inferred from the WG dataset analysis are congruent with earlier studies. Jansa et al. [9], using just the IRBP nuclear gene, estimated a date of 3-4 Mya for the origin of a clade containing *R. exulans*, *R. tanezumi *and, *R. preator *(sic). This is the equivalent of our 2.7 Mya split between the Asian and the Australo-Papuan clade (See Figure 1). When Jansa et al. [9] included both IRBP and cyt *b *data in their analysis the age of the nodes increased three fold and when cyt *b *alone was used the ages increased four fold. We had a similar problem with the 3G dataset which when analysed in BEAST, without the constraints described in the methods, returned dates that were almost three fold older than those returned by the WG dataset. It is worth noting also that we observed slightly older dates ~4.6 Mya (unpublished data) for the Asian/Australo-Papuan split on standard (ACGT) coding, compared with ~3.51 Mya obtained when we RY coded our 2008 [5] whole mitochondrial dataset. Rowe et al. [10] included three *Rattus *sp. in their analyses of the old endemic murines of the Sahul. They recovered evidence for the Asian/Australo-Papuan split with *R. leucopus *and *R. villosissimus *forming a sister pair relative to *R. norvegicus*. Although they did not report a chronogram, we can obtain a rough estimate of the age of the MRCA for *Rattus *from their Figure 4. The depth of the MRCA of *Rattus *was 26% of the depth of the closest dated node (B) in its ancestry (their Table 1), giving an estimated age of 2.52 (2.26 - 2.81) Mya. This date is concordant with our estimate of 2.7 Mya.

Our dates are also consistent with the earlier study of Robins et al. [5]. Whilst the node dates obtained from our WG BEAST analysis are all slightly younger than the equivalent ones from the previous study they are within the 95% credible intervals of the 2008 study. Since we have increased the sample size of the Australo-Papuan clade representation from one to eight the dates we report here are more reliable. These younger dates align more closely with those from the L1 (LINE-1, long interspersed repeated) retrotransposable elements study of Verneau et al. [24] who estimated the timing of several major speciation events within *Rattus sensu stricto *of which the two most recent are relevant to our study. The first at ~2.7 Mya gave rise to five lineages one of which led to *R. fuscipes *(the only representative of the Australian and New Guinean rats in their study) and another led to Asian *Rattus *species. This divergence is the equivalent of the split between our Asian and Australo-Papuan clades also dated at ~2.7 Mya.

The five lineage speciation events described by Verneau et al. [24] and the origin of the Australo-Papuan and Asian clades all estimated at ~2.7 Mya coincided with Pleistocene Ice Age events. The onset of the Pleistocene glaciation cycles was at about 3 Mya [50] and by 2.6 Mya the Pleistocene Ice Age was well established [51]. Major changes occurred in climate and available land in the region encompassing Island Southeast Asia, New Guinea and Australia. Such changes, for example at the highest glacial maxima New Guinea, Australia, and the islands of Misool, Waigeo, the Aru archipelago and Tasmania formed a single land mass[18], would have provided routes for dispersal and subsequently opportunities for speciation in *Rattus*. Also during the same time period, there was considerable dispersal of marsupials (bandicoots, dasyurids, phalangerid possums, and kangaroos) between New Guinea and Australia but diversification was more limited [52]. We can speculate that there was greater niche overlap between the newly migrated and endemic marsupials while *Rattus *was not so ecologically constrained. Glacial cycling with some 20 cycles in the last two million years continued to contribute to ongoing climate and sea level changes [17,50] and during this time radiation also continued in *Rattus*.

The more recent speciation events described by Verneau et al. [24] were a radiation in the Asian *Rattus *clade. Verneau et al's [24] study included 12 Asian *Rattus *species and four of these are represented in our study; *R. norvegicus, R. exulans R. rattus *and *R. tanezumi*. Their divergence estimates were in the time period 1.2 Mya to about 0.5 Mya. Our results are reasonably concordant with but probably more reliable than theirs because their analysis assumed a fixed rate molecular clock. In their L1 study the timing of the divergence of the *R. norvegicus *lineage at ~1.8 Mya compares with ~2.3 Mya in our study; *R. exulans *at ~1 Mya compares with our ~1.7 Mya and the divergence between *R. rattus *and *R. tanezumi *at 0.5 Mya compares with ours at ~0.3 Mya. The divergences in our Australo-Papuan clade occur over a similar time period (1.7 Mya - 0.5 Mya) as that in the Asian clade. *R. praetor *diverged at ~1.7 Mya, *R. leucopus *at ~1.6 Mya, *R. fuscipes *at ~1.4 Mya, *R. tunneyi *at ~1 Mya, *R. lutreolus *at ~0.9 Mya and the sister species *R. sordidus *and *R. villosissimus *at ~0.5 Mya. Despite the uncertainty in assigning the order of the deepest divergences in our Australo-Papuan clade, which is exacerbated by the relatively short time frame of ~0.2 My in which they occurred, the overall pattern from the WG and the 3G datasets suggests that the founding *Rattus *lineages reached New Guinea by at least 1.7 Mya and Australia by 1.4 Mya with further diversification occurring in both areas between ~1 - 0.5 Mya.

When sea levels were as little as 10 m below present land bridges would have connected New Guinea and Australia at the Torres Strait [53]. These land bridges which have occurred intermittently have also provided opportunities in more recent times for the reinvasion and subsequent isolation of rats between New Guinea and Australia in the Torres Strait area. In our phylogeny *R. sordidus *and *R. villosissimus *diverged most recently in the Australian *Rattus *clade. This position is consistent with an Australian origin of *R. sordidus*. In contrast the *R. leucopus *lineage diverged earlier than any of the native Australian rats thus supporting a New Guinean origin. The recent split between the Australian and New Guinean sister lineages at ~0.6 Mya suggests an invasion of Australia by *R. leucopus *followed by isolation. Our findings, therefore, support the Torres Strait faunal interchange hypothesis first put forward by Tate [15,16] with later support from Taylor and Horner [12], Taylor et al. [13] and Aplin [17].

## Conclusions

Although *Rattus *is a problematic genus and further investigations of the Australo-Papuan group are needed to resolve the relationships of the *Rattus *species of New Guinea we have clarified the pattern and timing of divergences among the Australian rats. Our whole mitochondrial genome analyses are concordant with Musser and Carleton's [1]*Rattus fuscipes *species group and resolve the positions of *R. fuscipes *and *R. lutreolus *within it. Our findings suggest that *Rattus *invaded Australia from New Guinea about 1.4 Mya and that this was followed by a period of rapid speciation. Our results also support and suggest a date of ~0.5 Mya for the hypothesized [15,16] more recent invasions across Torres Strait of *R. sordidus *into New Guinea and *R. leucopus *into Australia.

## Authors' contributions

JR and PM designed the project, planned and conducted lab work. All authors analysed data and contributed to the writing and editing of the manuscript. All authors approved the final manuscript.

## Supplementary Material

Additional file 1 **WG_partitioned.nxs**. The whole mitochondrial genome alignment partitioned into the three codon positions, and RNA stem and loop regions. Click here for file

Additional file 2 **3G_partitionedByRegion_RY_encoded.nxs**. The 3 gene regions (Cyt B, COI and D-loop) alignment partitioned into codon positions 1 and 2, codon position 3 as RY and all other positions. Click here for file

Additional file 3 **3G_partitioned.nxs**. The 3 gene regions alignment with each gene as a separate but unencoded partition. Click here for file

Additional file 4 **Sequence labels&accessions.xls**. Summary of sample information: sequence labels, species names and GenBank accessions. Click here for file

Additional file 5 **3G-WG_partitionedByRegion_RY_encoded.nxs**. The 3 gene regions alignment extracted from the specimens in the WG dataset. Click here for file

Additional file 6 **Rodent_cytb_full.nex**. The full alignment for the cytochrome *b *gene sequences of *Rattus *and closely related genera. Each sequence is labelled with its GenBank accession number followed by its species name. Click here for file

Additional file 7 **Rodent_cytb_full_tre.pdf**. Bayesian inference phylogram of the full cytochrome *b *dataset with the terminal nodes collapsed. Click here for file

Additional file 8 Rodent_cytb_full.tre Click here for file
