# Supplementary material for: Evolutionary relationships and divergence times among the native rats of Australia
Source: BMC Evol Biol. 2010 Dec 2;10:375. doi: 10.1186/1471-2148-10-375 (PMC3014932; doi:10.1186/1471-2148-10-375)
Supplement: Additional file 7 — Rodent_cytb_full_tre.pdf. Bayesian inference phylogram of the full cytochrome b dataset with the terminal nodes collapsed. [file 1471-2148-10-375-S7.PDF]

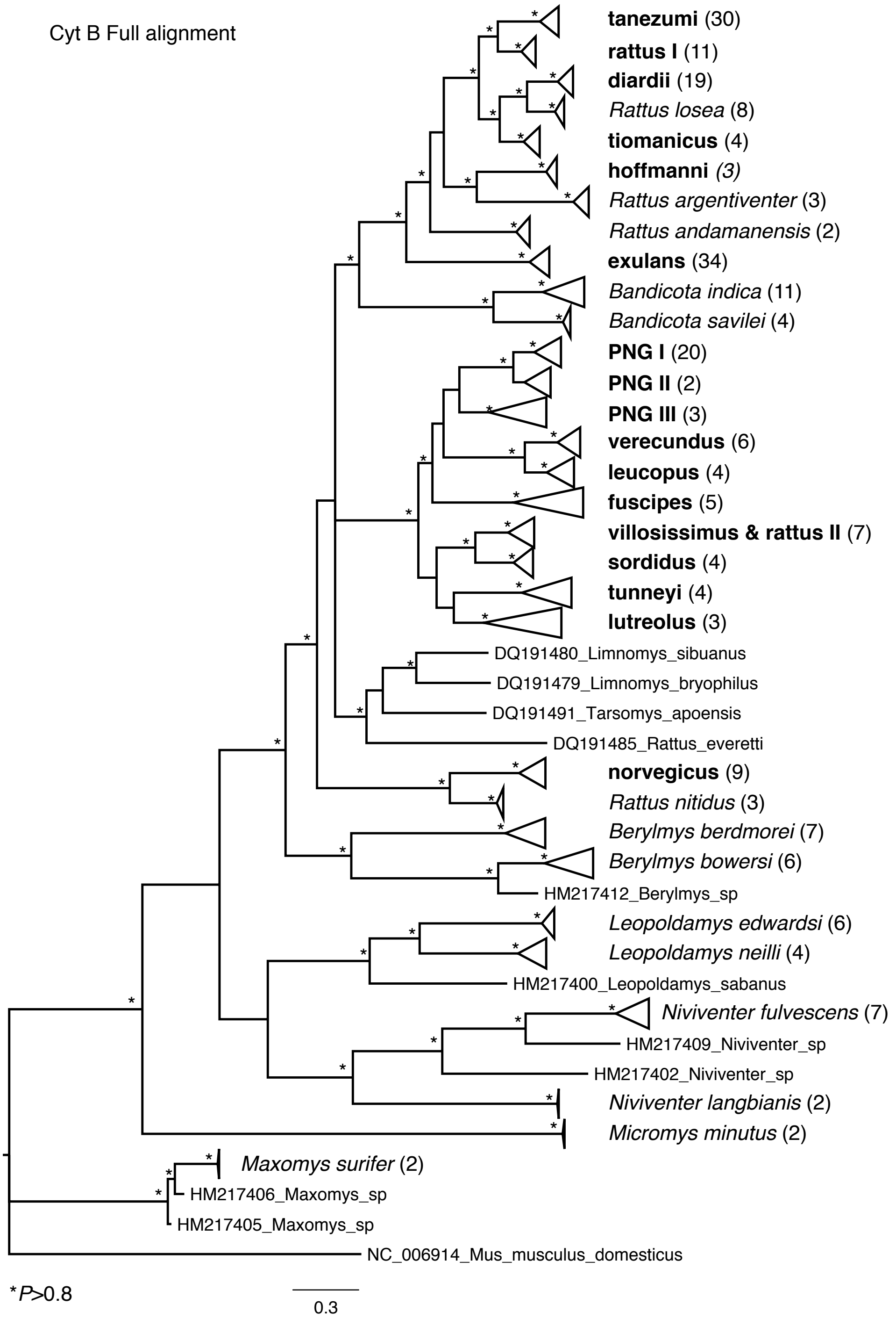

Phylogenetic tree of *Rattus sensu lato* estimated using Cyt B sequences from Genbank and this study(5_Rodent_cytb_full.nex). Groups labelled in boldface are clade names taken from [4], groups labelled with a binomial share the same species label but were not included in [4], and other sequences are labelled as the Genbank accession followed by the species name associated with the sample. The tree was estimated using Bayesian inference and clades with posterior probability > 0.8 are marked with *. The full tree is given as Additional file 7_Rodent_cytb_full.tre.
